# Cultivation of Kabul Dhingri (*Pleurotus eryngii*) mushroom by standardizing protocols in subtropical zones of world

**DOI:** 10.1038/s41598-021-94038-2

**Published:** 2021-07-19

**Authors:** Akansha Deora, S. S. Sharma, Poonam Kumari, Vinita Dahima, Suresh Kumar, M. Rohith

**Affiliations:** Department of Plant Pathology, Rajasthan College of Agriculture, Maharana Pratap University of Agriculture and Technology, Udaipur, Rajasthan 313001 India

**Keywords:** Microbiology, Plant sciences

## Abstract

The study is of great relevance with present day pandemic era where mushrooms have immunity enhancing properties and they convert agro-wastes into protein rich food. India is having a youth population of about 750 million and mushroom cultivation has good potential to contribute in national income as well as enhanced immunity. The key aspects undertaken during research were the spawn production, cultivation methodology, and the suitability of various factors affecting the production and yield attributes of *Pleurotus eryngii* under ambient conditions in subtropical areas. Study includes yield enhancing substrate, sterilization method, spawn and substrate quantity in the growing of King Oyster i.e. *Pleurotus eryngii* in subtropical zones. Paddy straw was found to be the best substrate giving the highest biological efficiency and producing maximum number of fruiting bodies which is otherwise burnt by farmers in India and it is a major cause of air pollution. Whereas, maize straw showed fastest spawn run and pin head emergence out of six tested substrates and supplements. But, due to the unavailability of paddy straw in this region, the other straws resulting in optimum yields are to be recommended. Chemical steeping of substrate with chlorine water at 0.4% + carbendazim at 2% + dichlorovos at 0.1% of water used for soaking showed best results in terms of biological efficiency whereas, water and aerated steam treatment of substrate at 85 °C-90°C for about 60–90 min supported the results in leaching of nutrients and thus, biological efficiency gets lower. Out of four spawn rates used, spawn rate of 5% was found significantly best resulting in maximum biological efficiency, fastest mycelial run, primordial initiation and highest average fruit body weight. All the experiments were found statistically significant except the experiment that was performed for evaluating the optimum quantity of substrate for bag preparation. There was not much difference in the obtained yields with respect to increase in the amounts of substrate. So, growing this mushroom from the obtained best results will result in better production with higher income even for the marginal farmers in subtropical zones of world.

## Introduction

The most cultivated mushroom globally is *Agaricus bisporus* (Button mushroom) followed by *Pleurotus sp.* (Dhingri mushroom) that constitutes about 27% of the world’s cultivated mushrooms^1^. *Agaricus bisporus* has prominently been cultivated in subtropical zones in winters and *Pleurotus sp.* is nowadays gaining importance. *Pleurotus eryngii* (DC.ex FR.) Quel. is still new to this area and hence, standardizing its cultivation protocols is necessary so that farmers can get good incomes and nutritive food at the same time at minimum inputs.

Mushrooms are large, fleshy and higher fungi that possess stalks and caps^2^. They are rich in nutrition and health beneficent substances and are good source of protein and fiber. Digestibility quotient is up to 95% in mushroom protein. The carbohydrate of mushroom is easily digestible and contains starch, pentose, hexose, disaccharides, amino sugars, sugar acids and sugar alcohols^3^. Edible mushrooms have very low lipid levels with high proportion of polyunsaturated fatty acids. They are low calorie food and because of fibers, mushrooms stay long in the stomach and they give a feeling of satiety which helps in diets^4^. Oyster mushroom falls under Phylum—*Basidiomycota*, Class—*Agaricomycetes*, Order—*Agaricales* and Family—*Pleurotaceae*^5^. *Pleurotus eryngii* is the largest species in the genus*, Pleurotus,* other species being *Pleurotus ostreatus, Pleurotus sajor-caju, Pleurotus florida*.

*Pleurotus eryngii* is also called as Kabul Dhingri and King Oyster mushroom because it is considered as the best of all *Pleurotus* species due to its splendid consistency of thick stem and cap and longer shelf life than any other Oyster mushrooms. Lower water content and firm flesh of the fruiting body may be the reasons that are making it best in quality^6,7^. It is a weak parasite that can grow on the roots or stem base of the live plants of the family *Apiaceae*^8,9^.

It is able to grow on a large variety of substrates. Cultivation of *Pleurotus eryngii* mushroom has been done on various agricultural and agro-industrial wastes in different parts of world including different sawdust, wheat straw, millet straw, soybean straw, cotton waste, peanut shells, sugarcane bagasse, wheat and rice bran^8,10–15^. It is a biotechnological process which is one of the most efficient in recycling the lignocellulosic organic wastes^16^.

Mushrooms as a whole are a great source of proteins (20–25%), polysaccharides (37–48%), fibers (13–24%), vitamins and minerals^17,18^ and has some secondary metabolites, including phenolics, polyketides, terpenes and steroids^19^.

It is a cholesterol-lowering dietary agent as it produces ergosterol and lanosterol and contains chemicals that boost the immune system and has anti-hypertensive, immune-modulating, antitumor, antibacterial, antioxidant, anti-hypocholesterolemic, anti-hyperglycemic, antiviral, antifungal, anti-inflammatory and anti-osteoporotic effects^20^.

## Results

### Effect of different substrates and supplements

**Figure 1:**
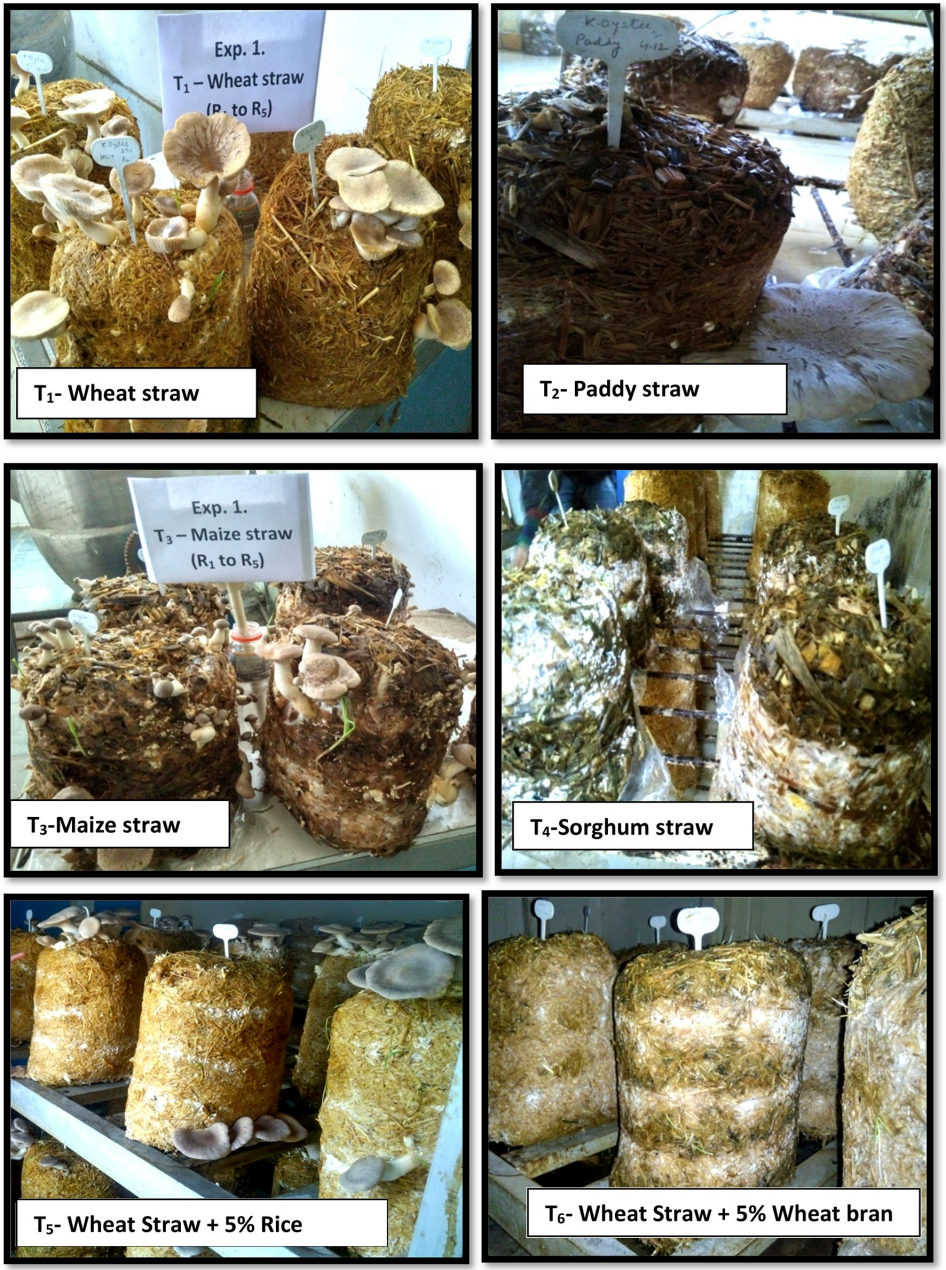
Photographsof different substrates and supplements used for *P. eryngii* production.

**Figure 2:**
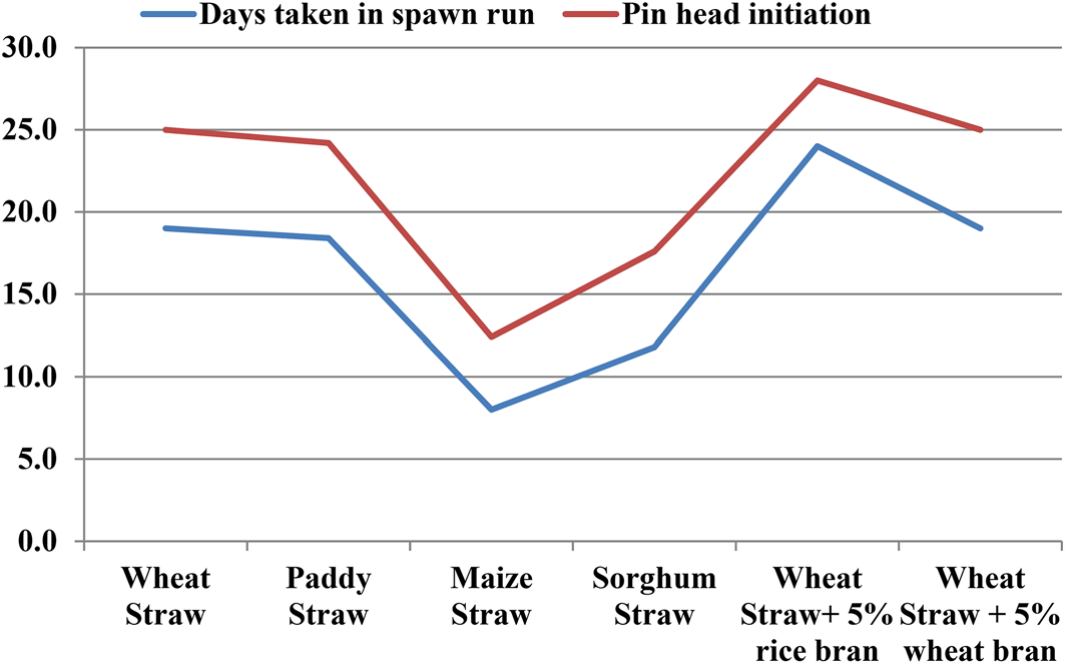
Average days taken in spawn run completion and days taken to pin head initiation on different substrates and supplements.

**Figure 3:**
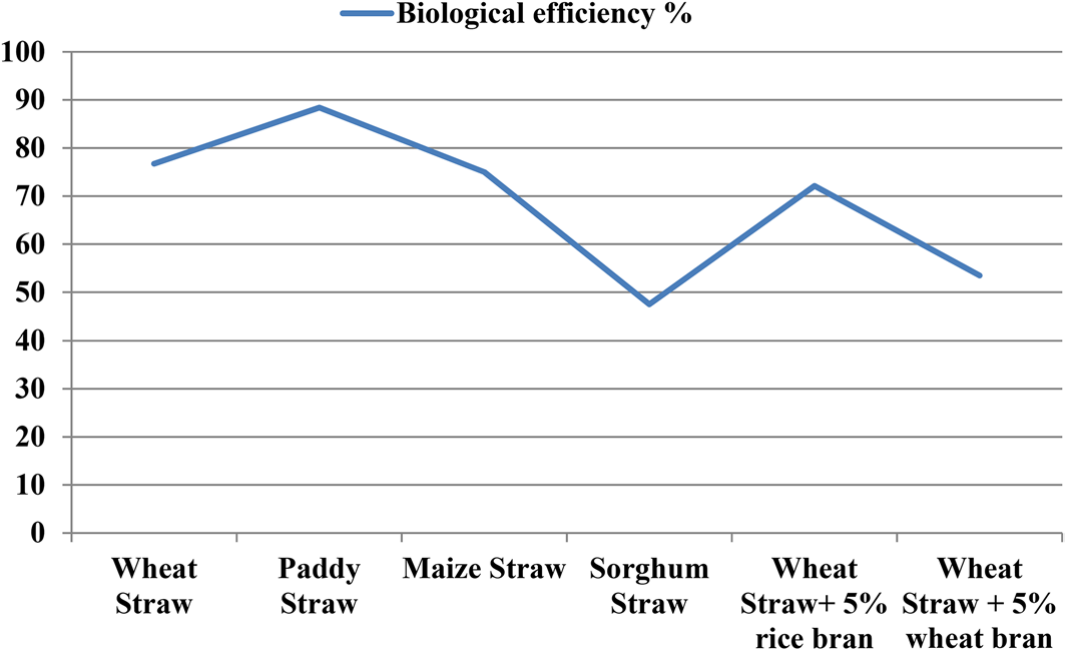
BE% obtained from different substrates and supplements.

Substrate is one of the essential factors involved in mushroom cultivation. Nature of substrate influences the production and quality of mushrooms in various aspects. So, we tested different substrates and supplements like wheat straw, paddy straw, maize straw, sorghum straw, wheat straw + 5% rice bran supplementation and wheat straw + 5% wheat bran supplementation to know the highest yielding substrate with minimum cost (Fig. 1). As per the data presented below in Table 1 and Fig. 2, the minimum time (8 days) taken by mycelium of *Pleurotus eryngii* to colonize the substrate completely was recorded on maize straw followed by sorghum straw (11.8 days). Maximum time (24 days) for spawn run completion was recorded on wheat straw supplemented with rice bran. The same trend was being followed with the time taken for initiation of pinheads. Minimum days for initiation of pinning were recorded on maize straw (12 days) and the longest duration was reported in case of wheat straw supplemented with rice bran (28 days). Contrary to this, maximum number of fruiting bodies (32.8) and the highest biological efficiency (88.4%) was obtained in case of paddy straw followed by wheat straw (76.7%) and maize straw (75%) (Fig. 3). Fruit body weight in case of *Pleurotus eryngii* reaches up to as high as 180–200 g with 12–14 cm broad pileus, 7–8 cm long and 5–6 cm thick stipe. Only averages are mentioned below in the table which were lowered by some underdeveloped fruit bodies raised due to lack of ideal management practices and by those fruit bodies which came out along with developed ones during harvesting due to their adjoined parts with developed fruit bodies. There was significant difference in yields of mushroom on these substrates and supplements.

**Table 1:** Effect of different substrates on spawn run, pinhead initiation, yield and morphology of *Pleurotus eryngii.*

Treatment	Days taken in spawn run	Days taken in Pin head initiation	Number of fruiting bodies	Yield (g)	Biological efficiency %	Average Fruit Body weight (g)	Fruit Body Measurement		
							Pileus (cm)	Stipe length (cm)	Stipe diameter (cm)
Wheat Straw	19.0	25.0	30.4	767.4	76.7	25.3	6.5	4.6	2.0
Paddy Straw	18.4	24.2	32.8	884.0	88.4	31.4	6.8	4.8	2.4
Maize Straw	8.0	12.4	30.2	750.0	75.0	26.7	6.2	4.3	1.5
Sorghum Straw	11.8	17.6	12.4	474.8	47.5	38.4	7.2	5.4	3.0
Wheat Straw + 5% Rice bran	24.0	28.0	17.8	721.0	72.1	55.1	7.9	5.0	3.0
Wheat Straw + 5% Wheat bran	19.0	25.0	9.4	534.8	53.5	96.4	9.0	5.0	3.8
SEm ±	0.4	0.5	3.5	28.2	2.8	17.5	0.2	0.1	0.1
CD(p = 0.05)	1.1	1.5	10.3	82.3	8.2	NS	0.6	0.4	0.3

SEm ± : Standard error of the mean.

CD(p = 0.05) : Critical difference, where p = level of significance.

All the observations are average of five replications.

### Effect of different sterilization methods

**Figure 4:**
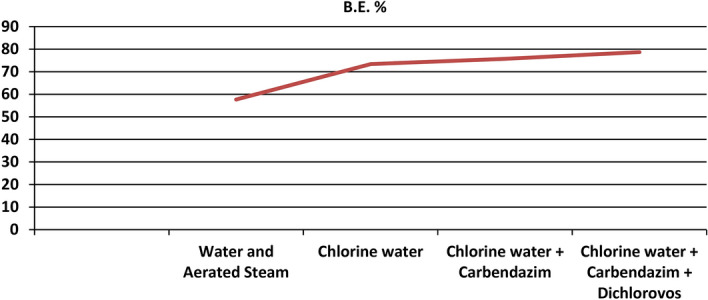
BE% obtained from substrates treated with different sterilization methods.

**Figure 5:**
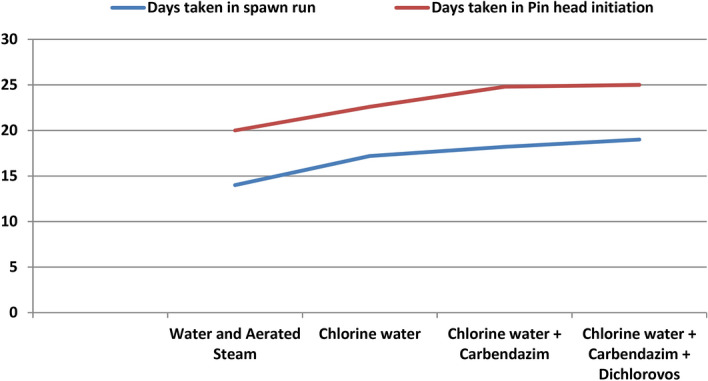
Average number of days taken in spawn run completion and average number of days taken for pin head initiation on substrates treated with different sterilization metho.

Sterilization of substrate is a crucial step during mushroom cultivation because it prevents pathogens like molds, bacteria, other fungi like *Coprinus*, *Aspergillus* etc. to colonize the substrate. We treated the wheat substrate (easily available) separately with different sterilization methods. On treating the wheat substrate with water and aerated steam treatment (WAST), yields got reduced when compared with other chemical treatments such as chemical steeping with chlorine water alone, steeping with chlorine water along with carbendazim and chemical steeping with chlorine water, carbendazim and dichlorovos altogether in fixed proportions. Chemical steeping of wheat substrate with chlorine water at 0.4% + carbendazim at 2% + dichlorovos at 0.1% of water used for soaking was found to be giving the highest yields (78.7%) while the lowest BE (57.7%) was obtained with WAST (Fig. 4). But, mycelial run completion (14 days) and initiation of pin heads (20 days) were observed to be fastest with WAST than chemical treatments. The reason might be the chemicals low down the vegetative growth of the fungus but, WAST results in leaching of the most of the nutrients. The experiment was statistically significant (Table 2, Fig. 5).

**Table 2:** Effect of different sterilization methods on spawn run, pinhead initiation, yield and morphology of *Pleurotus eryngii.*

Treatment	Days taken in spawn run	Days taken in Pin head initiation	Number of fruiting bodies	Yield (g)	B.E. %	Average fruit body weight (g)	Fruit Body Measurement		
							Pileus (cm)	Stipe length (cm)	Stipe diameter (cm)
Water and Aerated Steam	14.0	20.0	21.0	577.2	57.7	33.9	7.2	5.5	3.0
Chlorine water	17.2	22.6	25.8	734.0	73.4	31.4	6.9	5.4	2.2
Chlorine water + Carbendazim	18.2	24.8	27.8	757.4	75.7	32.5	6.8	5.3	2.0
Chlorine water + Carbendazim + Dichlorovos	19.0	25.0	30.6	787.4	78.7	27.2	6.5	4.6	2.0
SEm ±	0.3	0.4	3.3	39.7	4.0	5.8	0.1	0.1	0.1
CD(p = 0.05)	1.0	1.2	NS	119.0	11.9	NS	0.3	0.3	0.3

SEm ± : Standard error of the mean.

CD(p = 0.05) : Critical difference.

All the observations are average of five replications.

### Optimum Spawn rate

**Figure 6:**
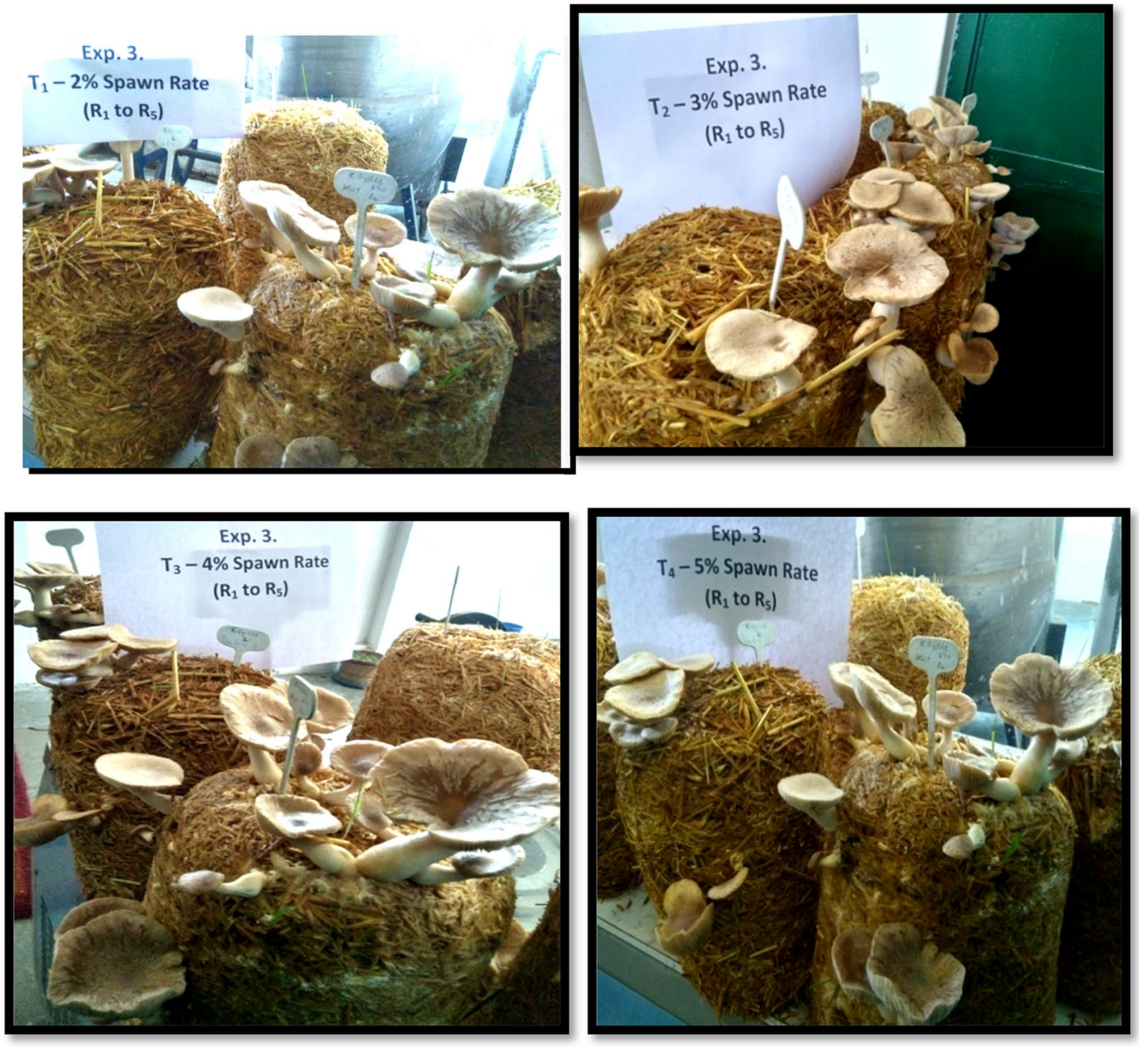
Photographs of different spawn rates for *P. eryngii* production.

**Figure 7:**
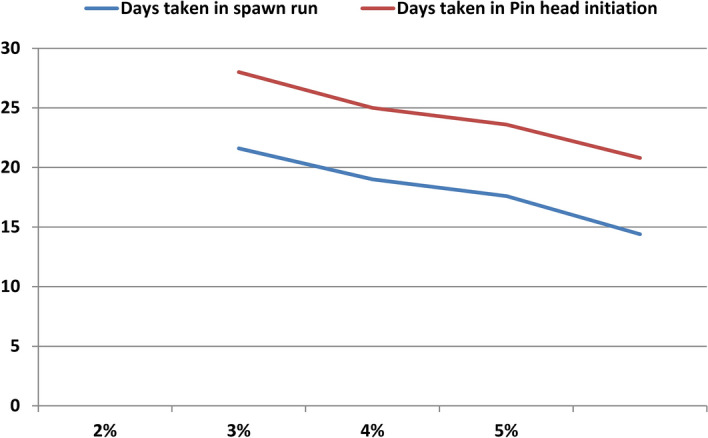
Average days taken in spawn run completion and average days taken to pin head initiation from different spawn rates.

**Figure 8:**
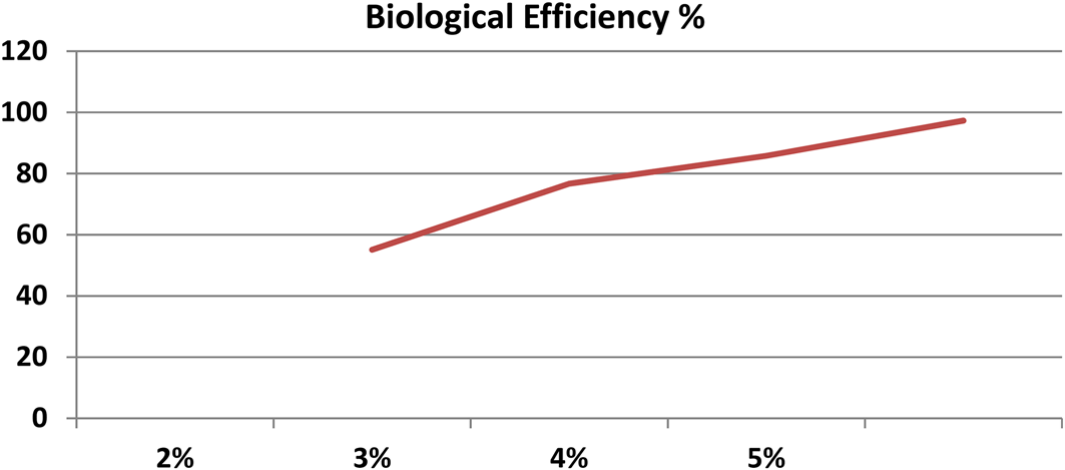
BE% obtained from different spawn rates.

Optimization of spawn rate is necessary to get high returns but at low cost. Different spawn rates (2%, 3%, 4% and 5% of wet substrate weight) were grown on wheat straw (Fig. 6). In this experiment, a clear cut trend is visible in the Table 3 and Fig. 7, with the increase in spawn dose, there was corresponding increase in the yield. The mean number of days taken for mycelial run completion and pinhead initiation of *P. eryngii* on wheat straw substrate from the date of spawning exhibited significant difference between different spawn doses. The spawn run got completed and pinheads first appeared with spawn rate of 5% (14.4 days and 20.8 days respectively), which proved to be the best spawn rate followed by 4% , 3% and 2% being lowest (21.6 days and 28 days respectively). Results for BE% are showing significant increment with the increased spawn amount up to a certain value. 5% of spawn rate gave the highest BE% of 97.3% and spawn rate of 2% gave the lowest BE% of 55.1% (Fig. 8). Temperature of substrate bags had already begun to increase at 5% spawn rate. So, we did not test spawn rate beyond 5%, because excess spawn would not give good economic returns, as the bags would be prone to high contamination due to temperature more than ambient value. The results were statistically significant.

**Table 3:** Effect of varied quantity spawn rates on spawn run, pinhead initiation, yield and morphology of *Pleurotus eryngii.*

Treatment	Days taken in spawn run	Days taken in Pin head initiation	Number of fruiting bodies	Yield (g)	Biological Efficiency %	Average fruit body weight (g)	Fruit Body Measurement		
							Pileus (cm)	Stipe length (cm)	Stipe diameter (cm)
2%	21.6	28.0	20.0	551.2	55.1	29.4	6.8	5.3	2.04
3%	19.0	25.0	30.6	767.4	76.7	26.0	6.5	4.6	2.02
4%	17.6	23.6	24.0	857.6	85.8	35.7	7.0	5.4	3
5%	14.4	20.8	23.8	973.4	97.3	41.6	7.9	5.0	3.03
SEm ±	0.3	0.5	1.4	34.4	3.4	3.1	0.1	0.2	2.52
CD(p = 0.05)	1.0	1.4	4.2	103.2	10.3	9.4	0.3	0.5	0.56

SEm ± : Standard error of the mean.

CD (p = 0.05) : Critical difference.

All the observations are average of five replications.

### Quantification of substrate to get optimum yield

**Figure 9:**
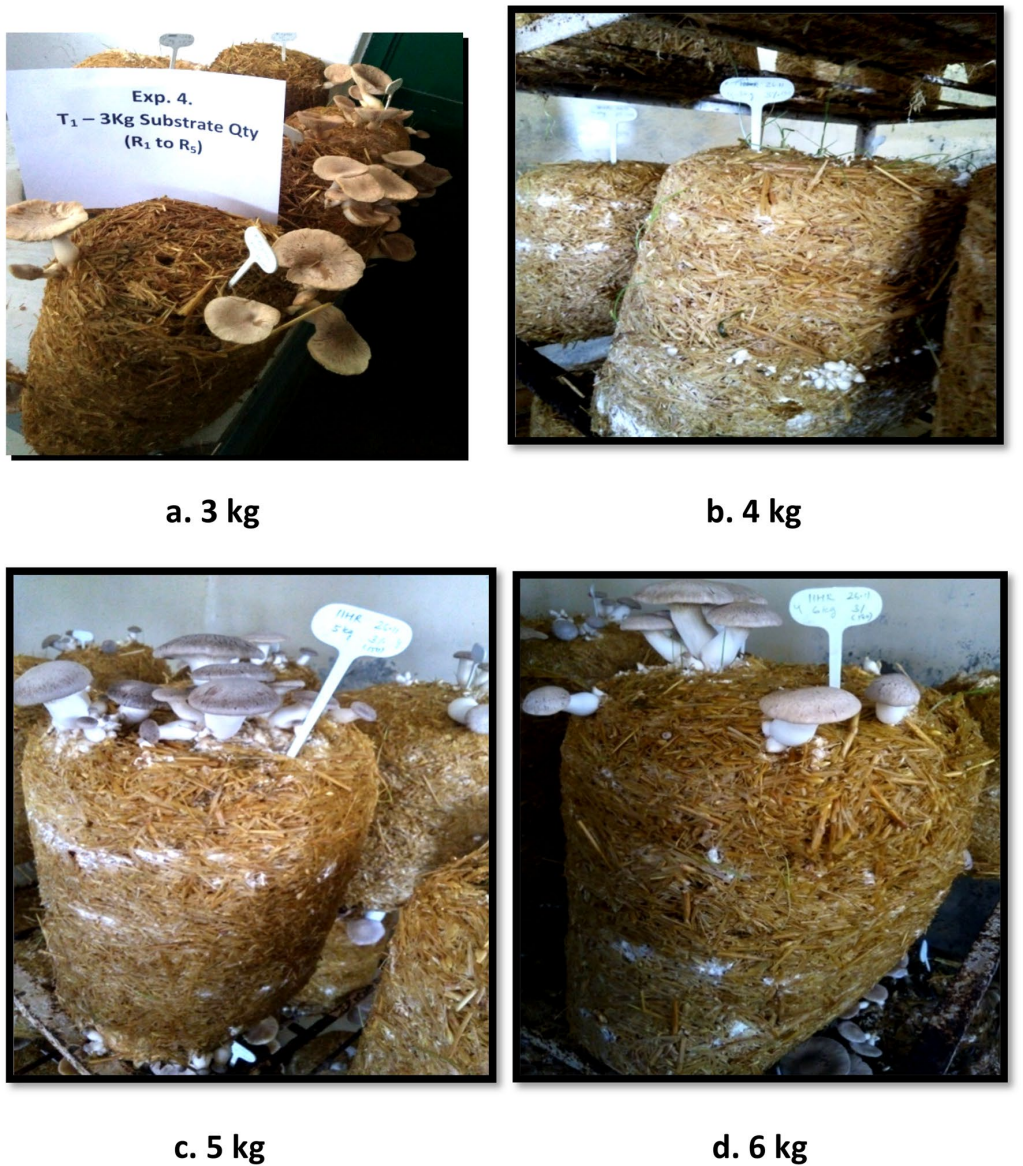
Photographs of different quantities of substrate for *P. eryngii* production.

**Figure 10:**
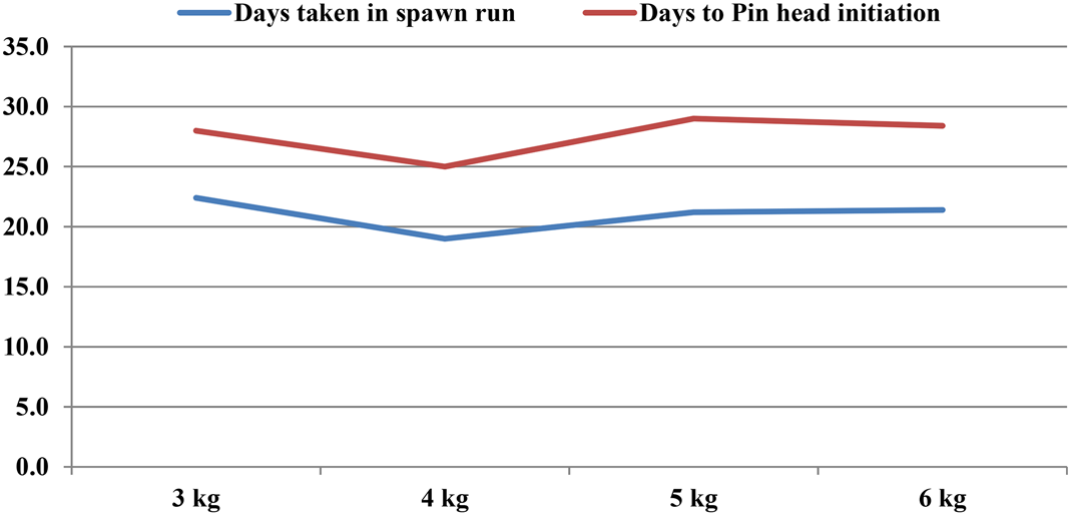
Average days taken in spawn run completion and days taken to pin head initiation on different substrate quantities.

**Figure 11:**
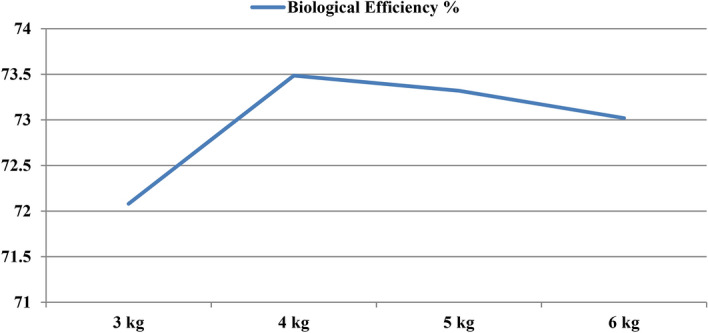
BE% obtained from different substrate quantities.

Substrate quantity has a practical importance in handling. We filled bags separately with 3 kg, 4 kg, 5 kg and 6 kg wheat straw on wet weight basis and spawning was done at 3% (Fig. 9). Data in Table 4 indicates that, there is no significant effect of increasing substrate quantity on the biological efficiency of *P.eryngii* unless the spawn rate is increased (Table 3). However, minimum days for spawn run completion and pin heads initiation (19 days and 25 days, respectively) were observed with 4 kg straw amount followed by 5 kg, 6 kg and 3 kg (Fig. 10). The treatments given for evaluating B.E. % and fruit body number, weight and fruit body measurement were statistically insignificant (Fig. 11). However, if we observe that there is an increase in yield per kg of substrate added, then it is lucrative to use larger bag, because the area occupied by a larger bag in the cropping room does not increase significantly. Hence, 5–6 kg bag of substrate would be worth adopting provided with proper care and management for cultivation.

**Table 4:** Effect of different substrate quantities on spawn run, pinhead initiation, yield and morphology of *Pleurotus eryngii.*

Treatment	Days taken in spawn run	Days taken in Pin head initiation	Number of fruiting bodies	Yield in (g)	Biological Efficiency %	Average fruit body weight (g)	Fruit Body Measurement		
							Cap diameter (cm)	Stipe length (cm)	Stipe diameter (cm)
3 kg	22.4	29.4	17.0	720.8	72.1	45.1	7.9	5.0	3.0
4 kg	19.0	25.0	19.6	979.8	73.5	52.3	8.0	5.2	3.1
5 kg	21.2	28.0	23.2	1222.0	73.3	52.1	8.4	5.1	3.5
6 kg	21.4	28.4	32.4	1460.4	73.0	56.2	8.0	5.3	3.0
SEm ±	0.3	0.3	4.2	98.3	6.2	7.1	0.2	0.1	0.1
CD(p = 0.05)	0.8	1.0	NS	294.6	NS	NS	NS	NS	0.3

SEm ± : Standard error of the mean.

CD(p = 0.05) : Critical difference.

All the observations are average of five replications.

## Discussion

In the current investigation, among the six tested substrates and supplements, paddy straw and wheat straw were found to be giving maximum yields followed by maize straw in climate of Udaipur, India (Table 1). Moreover, in India, paddy holds the first place among cereals production, thus, its straw can be widely used for mushroom cultivation at cheaper inputs. Wheat straw is also easily available in our country. Few also reported paddy straw as the best agro-waste material which took 12.6 days for spawn run completion and yield of 648 g/kg wet substrate as compared to wheat straw and sawdust^21^. Different *Pleurotus* spp. can be commonly cultivated on the pasteurized wheat or rice straw^22^. On cultivating *P.eryngii* taking mixture of wheat straw-cotton straw, wheat straw and millet straw supplemented with 15% rice bran, the highest BE being 73% was obtained with supplemented wheat, cotton straw^23^. Maize straw can also be suggested for Udaipur conditions, as it is a major crop here.

Quick spawn run can be attained by giving mere WAST to the wheat substrate but higher yields were produced by giving chemical treatments (Table 2), because the fungicides used to prevent the other saprophytes and fungal contamination like *Coprinus*, *Trichoderma*, *Aspergillus* and insecticides check insect attacks. Studies say that carbendazim treatment was found to be giving highest yields (BE: 106.93%) while the lowest BE with 75.83% was obtained with IHW (immersion in hot water). When treating substrate with IHW treatment, yields get reduced at least 20% when compared with other straw treatments such as steam treatment, chemical treatment or untreated wheat straw. The loss of these nutrients would be the reason of decrease in yield^24^.

Different doses of spawn also affect the B.E. % of Kabul Dhingri mushroom. The yield increase was directly proportional with the increase in spawn rate up to certain level (Table 3) because there is quick mycelial spread and less time would be needed for substrate colonization which prohibits other contaminants being infected and established in the straw. With further increase in spawn rate, rise in bag temperature was noticed. Researchers have also supported the view that increased amount of spawn increases the yield of *Pleurotus ostreatus* to a level^25^. Our studies with *Pleurotus eryngii* indicated that 5% spawn dose appeared superior from yield point of view. However, temperature of bags at this spawn rate too had started rising up. Temperature above than this would deteriorate the quality and yield of produce so higher rate of spawning was not preferred to get good economic returns. So, for the humid conditions in Udaipur region, 5% spawn rate is recommended. This is consistent with the findings from earlier experiments whose results indicated that spawn run was rapid when spawn dose was 5% in *P. sajor-caju* and *P. flabellatus*^26^.

Varying amount of straw substrate more or less affects the yield of mushroom. Out of different substrate weights (2 kg, 3 kg, 4 kg and 5 kg), 3 kg and 4 kg straw with 3% spawning rate gave the highest yield and biological efficiency (40%) than other treatments. Increase or decrease in amount of straw substrate resulted in low yield^27^. In our investigation for Udaipur conditions, the increase in substrate quantity has insignificant effect on the yield of *P.eryngii*. The reason might probably because surface area got increased with the increased straw amount and ultimately lowers down the spawn run completion period and as a result, it gets prone to contaminant fungi that directly affect the yield of mushroom.

## Conclusions

In our investigation, among different substrates and supplements, paddy straw and wheat straw were found to be the best followed by maize straw. In this region of Zone IV a *i.e*. Udaipur conditions, maize straw can also be suggested as it is a commonly growing crop suited to Udaipur climate. Thus, it becomes very easy for a farmer to grow mushrooms at the minimum cost by taking maize substrate from his own farm itself after chopping into fine pieces of about 1.5 cm length. The substrates may be useful in the production of a valued protein rich food. Cultivation of oyster mushroom on various agricultural residues offers economic initiatives for agribusiness to explore these residues as valuable resources and use them to produce protein rich mushroom products.

The reason of early spawn run in WAST is that substrate becomes free from saprophytes, but leaching of nutrients results in low production. So, complete chemical sterilization is recommended.

For the humid conditions in Udaipur region, 5% spawn rate is to be suggested because higher spawn rates leads to increase in substrate temperature which results into contamination of basal substrate. The increase in substrate quantity has insignificant effect on the yield of *P.eryngii* for Udaipur conditions.

## Methods

### Mother culture

The pure cultures of *Pleurotus eryngii* procured from different sources like Directorate of Mushroom Research, Chambaghat, Solan, Indian Institute of Horticultural Research, Hesarghatta, Bangluru and Mushroom Research and Training Centre, Maharana Pratap University of Horticulture, Karnal, Haryana were multiplied on malt extract agar and maintained in test tubes having two percent malt extract agar medium. The newly inoculated slants, thus formed were incubated at 20 ± 1 °C till the proper growth was obtained. From these three cultures, we have prepared six combinations through mycelial anastomosis. Among them, one strain was found to be the most suitable under the sub-tropical climate. We named it as “Pratap King Oyster-1” (P.K.O.-1) and this strain has been used in all the experiments that were performed at Rajasthan College of Agriculture, Maharana Pratap University of Agriculture and Technology, Udaipur, India.

### Preparation of master culture

Wheat grains were cleaned and soaked in water overnight and were boiled in water for 20–25 min. After drying, these seeds were mixed with 1% CaCO_3_ on wet grain weight basis to maintain the pH of grains at 7.0–8.0 as well as for avoiding the sticking and clumping of the grains. Properly mixed grains were then filled in milk glass bottles or polypropylene bags up to 2/3rd of capacity. These were sterilized in autoclave at 20 lbs psi pressure (126.5 °C) for 150 min. After cooling, these bottles/bags were inoculated by actively growing culture and incubated at 20 ± 1 °C in incubation room. After complete colonization the master spawn is ready for further use^6^.

### Preparation of substrate

The study complies with local and national regulations.

For the collection of seeds or plants, all relevant permits or permissions have been obtained. Wheat straw, paddy straw, maize straw, sorghum straw, wheat straw + 5% rice bran supplementation and wheat straw + 5% wheat bran supplementation were taken as treatments. Straws were chopped and soaked in fresh water for 24 h, along with chemical treatment to sterilize the substrate. Excess water from straw was drained off by spreading it on clean sloppy surface lined with polypropylene sheet or it can be kept on 150 mesh iron structure for decanting^27^.

Another experiment for testing the best substrate sterilization method was also laid out, in which the substrate was sterilized by different methods, viz*.,* WAST at 85–90 °C for 180 min, chemical steeping with chlorine water at 0.4%; chlorine water at 0.4% + carbendazim at 2%; and chlorine water at 0.4% + carbendazim at 2% + dichlorovos at 0.1% of water used for soaking. Out of them, the treatment resulting in the maximum biological efficiency was recommended. In this experiment, the wet weight of substrate was kept 3 kg (1 kg dry weight) with spawn rate being 3% of the wet weight of substrate. Quantification of substrates was also done in another experiment to obtain the standard substrate quantity which gives best results at low cost and can be recommended to the farmers. For this, bags were filled individually with 3 kg, 4 kg, 5 kg and 6 kg wheat straw on wet weight basis and spawning was done at 3%.

### Spawning

To know the best spawn rate, different spawn rates (2%, 3%, 4% and 5% of wet substrate weight) were taken and grown on wheat straw in individual bags with different spawn rates. Wet weight of substrate was kept 3 kg (dry weight was 1 kg) for each bag. Three layer spawning was done. Layer spawning means, the spawn is spread over substrate in three parts and after putting one third of wet substrate one part of spawn is spread and subsequently it is made in three layers and finally the bag is tied with rubber band.

### Crop management and picking

After 20–22 days of spawning, when the spawn run got completed and bags turned white due to completion of white mycelial run, the polythene covers were removed. Pin heads emerged out after 2–3 days of removal of polythene covers which later developed to form mature fruit bodies in 48–72 h^15^. At this stage, mushrooms were plucked off by twisting clock wise before they start shedding basidiospores.

### Biological efficiency

The biological efficiency was also calculated by using the given formula^2^.

$$\text{B.E}.\%= \frac{{\text{Weight of fresh fruiting bodies }}\left(\text{g}\right)}{{\text{Dry weight of substrate }}(\text{g})}\times 100$$

### Statistical analysis

Statistical Analysis Software (SAS software) developed by Indian Statistical Research Institute (ISRI), New Delhi was used for performing statistical analyses of data. The critical differences among treatment groups were determined using the ANOVA test at level of significance (p = 0.05). All the experiments were carried out in five replications in Completely Randomized Design (CRD).

The data analysis for this paper was generated using SAS software. Copyright [2014] SAS Institute Inc. SAS and all other SAS Institute Inc. product or service names are registered trademarks or trademarks of SAS Institute Inc., Cary, NC, USA.

## Data Availability

Requests for data and materials should be addressed to S.S.S (email: sharmass112@gmail.com).
